# Fertility-Sparing Surgery in Early Epithelial Ovarian Cancer: A Viable Option?

**DOI:** 10.1155/2012/238061

**Published:** 2012-02-23

**Authors:** Christina Fotopoulou, Ioana Braicu, Jalid Sehouli

**Affiliations:** Department of Gynecology, European Competence Center for Ovarian Cancer, Campus Virchow Clinic, Charité University Hospital, 13353 Berlin, Germany

## Abstract

Epithelial ovarian cancer (EOC) continues to represent one of the most lethal conditions in women in the western countries. With the shifting of childbearing towards higher age, EOC increasingly affects women with active childbearing wish, resulting in major impacts on treatment management. Next to the optimal therapeutic treatment strategies, gynecologic oncologists are being asked to incorporate into their decision-making processes the patients' wish for fertility preserving alternatives ideally without compromising oncologic safety. Nowadays, fertility-sparing surgery represents an effective alternative to conventional radical cytoreduction in younger women with early stages of the disease. As such, this paper considers indications for fertility sparing surgery in EOC, reflects on outcomes from the oncologic and reproductive data of the largest and most relevant series outcomes data, reporting on fertility sparing techniques in EOC, reviews medicamentous efforts to prevent chemotherapy induced gonadotoxicity, and discusses future aspects in the gynecologic cancer management.

## 1. Introduction

Epithelial ovarian cancer (EOC) continues to represent a lethal condition which commonly affects women in a multifocal and peritoneal metastasized fashion. Attributed to a special tumor biology and large heterogeneity, clinical outcomes in EOC vary broadly and although significantly associated with an adequate systemic and operative treatment, they often detach themselves from these common and known influential factors and follow not yet fully understood tumor biology patterns [1].

With the constant shifting of childbearing age towards higher ages, the increasing incidence of EOC in women with active childbearing potential constitutes a therapeutic dilemma [2]. Both patients and treating physicians are being encountered with the abrupt loss of childbearing potential due to the malignant disease, while alternatives are being sought that try to preserve a last hope of fertility within the antitumor treatment [3].

Even though radical surgery with the primary objective of maximal tumor reduction is currently the established cornerstone in the management of advanced EOC [4], fertility-sparing techniques are being increasingly incorporated in the therapeutic strategies in early or not bulky forms of the disease. Equivalent to the nowadays established organ-preserving techniques in borderline ovarian tumors, also in early EOC, strategies like preservation of the contralateral ovary and uterus or even in highly specialized cases peritonectomy of the pelvis and uterus serosa to avoid the need of hysterectomy are being recruited [5, 6]. The subsequent systemic chemotherapy within “fertility protecting” programs where chemotherapy is being applied under the concomitant ovarian protection via substances like GnRH analog a additionally induce fertility-sparing treatment and offer the affected woman a hope for conception after completion of the anticancer treatment.

Nevertheless, the inevitable question arises, whether fertility-sparing surgery (FSS) for EOC harbors life-threatening risks, which compromise patient's survival and set any chance for a normal family life into the background due to early and potentially chemotherapy-resistant relapse.

Since no randomized trials exist or will ever exist to prospectively evaluate and answer this question, our experience is limited to scattered, retrospective cases series. The aim of this paper is to provide an overview of the most significant hitherto reports and discuss future perspectives.

## 2. Current Indications for FSS in EOC

FSS for ovarian neoplasms have been traditionally adopted in early-stage malignant ovarian germ cell tumors and in ovarian sex cord-stromal tumors such as granulosa-cell tumors, Sertoli-Leydig cell tumors, ovarian dysgerminomas, as well as in borderline tumors of the ovary with excellent reproductive outcomes without compromising oncologic safety [7–9]. Gold standard remains however hereby an adequate operative staging in order to unmask occult advanced disease with therapeutic consequences and impact on overall prognosis.

For invasive EOC data are much more constricted and limited to retrospective, nonrandomised series referring mainly to patients with low-grade stage IA tumors with favorable histology, while data regarding higher stages of the disease or unfavorable constellation of histologic characteristics are rather conflicting [6, 10–12]. Overall, in selecting optimal candidates for FSS, the amount of evidence has been too small to accurately estimate the risk of leaving a microscopic tumor in the contralateral ovary [13], especially in high-grade disease with positive peritoneal cytology.

According to the 2007 guidelines of the American College of Obstetrics and Gynecology (ACOG), fertility-sparing surgery for reproductive-age patients with invasive EOC is recommended for highly or moderately differentiated stage IA disease with non-clear-cell histology [14]. In an equivalent manner, the European Society for Medical Oncology (ESMO) referring in 2008 to fertility-sparing techniques in EOC identified patients with unilateral stage I tumor without dense adhesions showing favorable histology (i.e., high or moderate differentiated, non-clear-cell histology) as being the optimal candidates for this procedure [15]. However, the number of published studies concerning fertility-sparing surgery in young EOC patients is rather limited and the evaluated patient's samples too small to allow unanimous consensus regarding the definition of the selection criteria of the optimal candidate for fertility-sparing surgery in stage I EOC. That leads to a broad variety of national guidelines regarding FSS worldwide, especially in respect to Ic (iatrogenic versus not) and/or poorly differentiated disease and to clear cell histologic subtype [16]. Moreover, under additional consideration of the relatively recently emerging dualistic model theory of EOC pathogenesis, which divides EOC in type I and type II disease [17], patients selection for FSS could theoretically be performed under this perspective and hence indicated for early-stage, type I tumors, even though there is currently no evidence that would support such an approach.

Open questions remain if there should be differentiation between iatrogenic Ic disease due to intraoperative tumor rupture versus Ic due to tumor on ovarian surface or malignant cells in peritoneal cytology, whether patients with G3 tumors with no evidence of further metastatic disease in adequate staging are eligible of FSS and whether nonserous or non-endometrioid histologic subtypes should be a priori excluded from any organ-preserving technique.

## 3. Oncologic Outcomes after FSS in EOC

Data from the largest report series in the literature concerning oncologic outcome after FSS in EOC are summarized in Table 1. Zanetta et al. published in the year 1997 one of the first reports regarding this issue and was the first to systematically show that fertility-sparing surgery is a safe treatment option for early-stage patients with acceptable oncologic safety profile [12]. Various case series or case reports followed since then with a large peak in the last two years of reports mostly originating from Japan. Satoh et al. attempted for the first time in 2010 to systematically determine selection criteria for fertility-sparing surgery in stage I EOC on the basis of clinical outcomes of more than 200 stage I EOC patients who underwent fertility-sparing surgery [16]. A relapse rate as high as 8.5% was reported with 27% of the relapsed patients presenting recurrence exclusively in the remaining ovary without any distant or peritoneal metastases. Hence, the authors identified stage Ia EOC patients with favorable histology, that is, mucinous, serous, endometrioid, or mixed histology and grade 1 or 2, as the optimal candidates to safely undergo fertility-sparing surgery even without an obligatory subsequent platinum-based adjuvant chemotherapy. In case of stage Ia disease with clear cell histology or stage Ic with unilateral ovarian involvement and favorable histology, authors emphasized the need of a complete surgical staging and an adjuvant platinum-based chemotherapy, since the 5-year recurrent-free survival rate of the fifteen evaluated patients with stage Ic clear cell carcinoma was with 66.0% comparably high, while the fifteen patients with stage Ia clear cell carcinoma showed no evidence of local or distant recurrence. Concerning patients with unfavorable constellation of histological tumor type, that is, stage Ia/G3 disease or stage Ic with clear cell or G3 histology, the authors recommended their exclusion from any fertility-sparing surgical approach [16]. 

When collectively evaluating most published results so far, mean relapse rates are estimated to be around 10%, even in patient's cohorts which included also Ic stage disease [12, 13, 18, 20–23]. Nevertheless, when accurately examining the characteristics of the patients who suffered from relapse, they belonged mainly to the subgroup with Ic and/or G3 tumors. Interestingly in many studies no differentiation occurs between Ic due to iatrogenic cyst rupture versus Ic due to malignant cells in the douglas cytology or surface involvement. Kajiyama et al. [23] assessed survival after FSS separately for these two patients' subgroups, that is, iatrogenic versus tumorbiologic Ic. He came to the conclusion that progression and overall survival of the patients with stage Ic (surface involvement/positive cytology) were significantly poorer than those of patients with stage Ia after FSS, whereas this was not the case when comparing survival rates of patients after FSS with Ia versus iatrogenic Ic disease, that is, after intraoperative tumor rupture.

In the study of Zanetta et al., none of the women undergoing bilateral oophorectomy had microscopic foci of cancer in the normal-looking contralateral ovary. The rate of bilaterality is congruent with the previous observations of other authors who reported an extremely low rate of contralateral involvement for mucinous tumours [12].

In two very recent studies originating also from the same authors group by Kajiyama et al., the authors assessed the feasibility of fertility-sparing surgery in patients with clear cell or mucinous carcinoma of the ovary, two histological types which have been associated in various reports with a rather less favorable prognosis [16, 22]. In both analyses the authors concluded that FSS in presence of these two histological subtypes was not necessarily associated with a poorer prognosis than after radical surgery and hence feasible. Data are shown in Table 1. This can be possibly attributed to the fact that the negative impact of unfavorable histology on survival has mainly been established for advanced-stage disease III and IV [24, 25]. Underlying theories are mainly based on an increased chemotherapy resistance to the conventional carboplatin and paclitaxel regimens of mainly mucinous cancers resulting in emerging attempts to treat these types similar to intestinal cancers with oxaliplatin and capecitabine ± bevacizumab within randomized trials [24]. In early stage Ia, however, no adjuvant treatment is necessary, with complete resection and adequate staging consisting a sufficient treatment plan.

When attempting to identify a recurring profile of the patients who relapsed after FSS, then no specific pattern can be identified, since in various series also Ia patients tend to recur, whereby here one has to consider the possibility of occult more advanced disease after inadequate staging. One of the major limitations of the existing studies is that not all of the cases underwent an obligatory systematic lymphadenectomy, in which case there might occur an up-staging of some patients [10, 16]. Reported death rates are ranging between 2 and 15%.

## 4. Reproductive Outcomes after Fertility-Sparing Surgery in EOC

The issue of impaired fertility in young cancer survivors represents a therapeutic dilemma for both treating physicians and affected patients. Systemic and operative oncologic treatments consistently compromise the ovarian function, often resulting in infertility and premature menopause [26–28].

Data regarding reproductive outcome after FSS in EOC are summarized in Table 2. Literature data concerning the rate of women with successful conception after FSS accounts approximately 30% of all patients however, if one takes into consideration only the women with childbearing wish who actively tried to conceive, then rates of successful conception are substantially higher and range from 66% to 100%, indicating that no relevant reproductive impairment exists after FSS. Also, where reported, only the minority of patients required assisted reproductive techniques for a successful conception and pregnancy [3, 10–12, 16, 18, 29].

When evaluating the incidence of spontaneous abortions, then rates range between 11% and 33%. No evidence is however given regarding details about the gestational week, the onset of symptoms, and whether there was a habitual recurring modus, so that no conclusions can be extracted about the cause of the abortions and their pathophysiology.

In the recent evaluation by Satoh et al. [16], there are even details given regarding the restoration of the menstrual cycle after completion of oncologic treatment. A hundred and eighty two patients (96.8%) of the overall 188 who gave information on their menstruation had almost the same cycle of menstruation as before treatment. However, six (5.0%) of the 121 patients who received platinum-based chemotherapy presented a persistent secondary amenorrhea up to 224 months after completion of 4–6 cycles of systemic chemotherapy. Details about the reproductive outcome of this population are given in Table 2. Five (9.1%) of the 55 patients who successfully conceived have been stated to receive an infertility treatment before pregnancy. Interestingly, the authors report four (9.4%) of the 53 patients who gave birth to children having underwent completion surgery after childbearing, consisting of hysterectomy and contralateral salpingo-oophorectomy.

Wherever reported, none of the patients who successfully conceived and gave birth presented any relevant, cancer-related clinical problems during the perinatal period. Also no higher rates of congenital malformations or abnormal fetal outcomes have been reported in the current literature [11, 16, 32].

## 5. Hormonal Support Options for Ovarian Protection in Chemotherapy-Induced Gonadotoxicity

It is well known that the number of oocytes decreases as a normal process from the fetal life up to menopause. At 20 weeks of gestation, female infants have about six to seven million oocytes, newborns only one to two million, and women at the age of 37 have only about 25,000 oocytes left. Chemotherapeutic agents, which act cytotoxic by interrupting the normal cell cycle and inducing apoptosis do also negatively affect the highly endocrine-active ovarian tissue. Cisplatin and its analogues, that is, agents which play a highly significant role in the management of ovarian cancer, have been proven to present a risk factor for ovarian failure by an estimated odds ratio as high as 1.77 [28]. In order to decrease chemotherapy-induced gonadotoxicity on young women after FSS, hormonal protection is being recruited to force the ovarian tissue by pituitary downregulation to enter into a state of inactivity and so to make it less susceptible to cytotoxic agents. Various agents have been applied such as GnRH agonists or antagonists, oral contraceptives, or even in a newly setting the selective estradiol receptor tamoxifen. The protective effect of GnRHa against chemotherapy-induced gonadotoxicity is still under debate [33]. The reason is mainly the lacking of large prospectively evaluated randomized studies to confirm and establish the protective effect of such agents. In a recent phase II study by the German Hodgkin Study Group, young women with advanced-stage Hodgkin lymphoma were randomly assigned either to receive daily OC or monthly GnRH-a during escalated combination therapy with bleomycin, etoposide, adriamycin, cyclophosphamide, vincristine, procarbazine, and prednisone (BEACOPPesc). The trial had to close prematurely after an interim analysis which showed that in both arms the ovarian follicle preservation rate was 0% with a 95% confidence interval ranging between 0% and 12% [34]. To demonstrate the protective effect of the GnRH analogue triptorelin, on chemotherapy-induced ovarian gonadotoxicity, Tan et al. applied different doses of triptorelin in combination with the alkylating agent busulfan in sexually mature, virgin, female mice. Results have demonstrated a dose-dependent protective effect against gonadotoxic chemotherapy of a GnRH analogue on ovarian reserve, thus suggesting a novel application of GnRH analogues in fertility preservation [35]. Almost all clinical studies assessing the protective effect of GnRH agents have been conducted in patients with haematologic malignancies, breast cancer, or even nonmalignant diseases which however require a cytotoxic treatment like lupus erythematodes [36]. Reviewing of all published studies using GnRH-a or oral contraceptives, data clearly demonstrate that they are too limited to provide conclusive statistical evidence concerning the reduction of premature ovarian failure, even though most studies analyzing the effect of hormonal protection during chemotherapy have shown a reduction of POF in patients receiving GnRH-a or OC during systemic chemotherapy [36]. Recently, also the combination of GnRH antagonist and analogue has been recruited to reduce time for pituitary downregulation before chemotherapy and to avoid delayed cancer treatment [33]. Even though there are no sufficient data to support this strategy to date, this combination approach mainly plays a role in haematologic malignancies where treatment has to be initiated within days; in the case of completely staged early EOC, a delay of days or 1-2 weeks does not have a tremendous impact on survival.

## 6. Future Perspectives

The possibility of reproductive dysfunction as a consequence of cancer treatment has an established negative impact on the quality of life of cancer survivors [37]. The merged role of the treating physician as both life-save and protector-of-future fertility has made the field of oncofertility a substantial part of gynecologic oncology nowadays. Since prospectively designed, randomized trials to evaluate safety of FSS are neither possible nor ethical maintainable, our experience in outcomes after FSS mainly originates from retrospective case series. The gynecologic oncologist is in a unique position to provide young cancer patients with up-to-date fertility preservation information and fertility-sparing surgical alternatives [37]. However, even after FSS, many young patients fail to fulfill their childbearing wish, due to various reasons, such as early relapse in the remaining ovarian tissue or due to diminished ovarian reserve. Here additional techniques like cryopreservation of ovarian tissue, oocytes, or even embryos are called to offer an additional safety option to young women. A considerable pitfall however in this approach is the potential risk that the cryopreserved ovarian tissue might harbor malignant cells that could induce disease recurrence. Furthermore, in case of cryopreservation of ovarian tissue alone, described rates of successful pregnancies are low. Future perspectives lie hence in further broadening and optimizing this option by improving efficacy through maximal exploiting of the stored ovarian tissue and increasing oncologic safety. There are novel studies which examine the possible presence of malignant cells in ovarian cortex from patients with ovarian tumors by xenografting of the ovarian tissue into severe combined immunodeficiency mice. The group around Lotz et al. could recently show that none of these mice presented symptoms of reintroduced malignancy after xenografting of the ovarian tissue of patients with malignant ovarian tumors. Moreover, microscopic and immunohistochemical evaluation of the grafts did not raise any suspicion of residual malignant disease [38]. Xenografting in the murine back muscle is being currently further explored as a method for human ovarian tissue transplantation [39]. As a distant dream appears, also the possibility of isolating healthy ovarian tissue or oocytes of bilateral tumor affected ovaries, where an ovarian preserving approach appears highly hazardous.

## 7. Conclusion

After thorough insight of the current literature, FSS in early-stage EOC appears an absolutely viable and safe option for women younger than 40 years who wish to preserve their childbearing potential after careful consideration of histologic subtypes. The optimal indication is referring to stage Ia G1/G2 disease, as well as stage Ic with favorable, that is, non-clear-cell histology. Here there has to be differentiated between iatrogenic—due to intraoperative tumor rupture—versus biologic Ic disease—due to surface involvement or positive Douglas cytology—since the latter is associated with less favorable outcomes after FSS. In case of stage Ic disease and clear cell histology, there is increasing evidence of a poorer relapse-free survival compared to non-clear-cell histology. For that reason a fertility-sparing approach in this special patients collective should be indicated only after thorough discussion and informed consent of the affected patients with careful balancing of the risks and benefits. In any case, the treating gynecologic oncologist should be fully aware of his double role in treating the malignant disease as well as in providing oncofertility care to young EOC patients, by considering offering fertility-sparing alternatives when allowed so by tumor stage and histologic differentiation.

## Figures and Tables

**Table 1:** Relevant case series reports in the literature concerning fertility-sparing surgery in epithelial ovarian cancer: oncologic outcome.

Author [reference]	Pts *N*	Median Age (y)	FIGO stage *N* (%)	Grade *N* (%)	Histology *N* (%)	Relapse rate *N* (%)	Mean PFS (mo) or 5 y DFS	Characteristics of pts who relapsed	5 y OS	Death	LND
Kwon et al. 2009 [18]	21	26.7	17 (81%) IA 4 (19%) IC	16 (76%) G1 3 (14%) G2 2 (9.5%) G3	16 (76%) muc 2 (9.5%) endom 2 (9.5%) clear cell 1 (4.7%) serous	1 (4.7%)	34	IC, muc	NR	NR	Yes
Kajiyama et al. 2008 [19]	10	35.9	4 (40%) IA 6 (60%) IC	NR	10 (100%) clear cell	1 (10%)	33	IC, G2, clear cell	NR	1 (10%)	Optional
Schlaerth et al. 2009 [3]	20	27	11 (55%) IA 9 (45%) IC	14 (70%) G1 5 (25%) G2 1 (5%) G3	11 (55%) muc 1 (5%) serous 6 (30%) endom 1 (5%) clear cell	3 (15%)	4.3 (9–22)	2 IC, 1 IA	84%	3 (15%)	Yes
Borgfeldt et al. 2007 [20]	11	NO	IA IC	G1 G3	NR	1 (9%)	NR	IC, G3	NR	NR	Yes
Schilder et al. 2002 [11]	52	26	42 (81%) IA 10 (19%) IC	38 (73%) G1 9 (17%) G2 5 (9.6%) G3	25 (48%) muc 10 (19%) serous 5 (9.6%) clear cell 2 (4%) mixed	5 (9.6%)	14 (8–78)	4 IA, G1 1 IC, G2 2 muc 2 serous 1 endom	98%	2 (4%)	Yes
Kajiyama et al. 2011 [21]	74	<40	36 (48%) Ia 1 (1.3%) Ib 37 (50%) Ic	57 (77%) G1/G2 4 (5.4%) G3	4 (5.4%) serous 43 (58%) muc 13 (18%) clear cell 4 (5.4%) endom	NR	87.9% 5 y DFS	NR	90.8%	2 (2.7%)	Yes
Zanetta et al. 1997 [12]	56	29	32 (57%) : IA 2 (3.5%) : IB 22 (39%) : IC	35 (62%) G1 14 (25%) G2 7 (12%) G3	18 (32%) serous 23 (41%) muc 13 (23%) endom	5 (9%)	NR	2 IA,G2, endom 1 IA, G3, muc 1 IA, G1, serous 1 IC, G2, serous	NR	4 (7%)	Yes
Satoh et al. 2010 [16]	211	29	126 (60%) IA 85 (40%) IC	G1: 160 (76%) G2: 15 (7%) G3 6 (2.8%) clear cell: 30 (14.2%)	126 (60%) muc 27 (13%) serous 27 (13%) endom 30 (14.2%) Clear cell	18 (8.5%)	33.3–100% 5 y DFS	Mostly Ic,G3 Ia, G3 Ic, clear cell	66.7–100%	5 (2.4%)	Yes
Kajiyama et al. 2011 [13]	41	<40 y	27 (66%) : IA 14 (34%) : IC	NR	100% muc	3 (7.3%)	90.5%		97.3%	1 (2.5%)	Yes
Kajiyama et al. 2011 [22]	80	35	40 (50%) IA 40 (50%) IC	—	45 (56%) muc 3 (3.75%)serous 15 (19%) endom 16 (20%) clear cell	10 (12.5%)	85.5%–92.9% 5 y DFS	2: IA 8: IC	89.3%– 90.5%	0	Yes
Kajiyama et al. 2010 [23]	60	30	IA 30 (50%) IB 1 (1.7%) IC 29 (48%)	41 (68%) G1 7 (12%) G2 2 (3.3%) G3	Serous 5 (8.3%) Muc. 34 (56.7%) Endom 11 (18 %) Clear cell 10 (17%)	8 (13%)	89.8%	2 IA, 1 IB 5 IC	89.8%	0	

Total	580	26–35.9	333 (57%) IA 4 (0.7%) IB 234 (40%) IC	334 (57%) G1 39 (6.7%) G2 20 (3.4%) G3	300 (52%) muc 51 (9%) serous 128 (22%) clear cell 65 (11%) endom	50 (8.6%)	33.3–100% 5 y DFS		66.7–100%	18 (3.1%)	Yes

muc: mucinous; endom: endometrioid.

**Table 2:** Relevant case series reports in the literature concerning fertility-sparing surgery in epithelial ovarian cancer: reproductive outcome.

Author [reference]	Patients (*N*)	Mean age (years)	FIGO stage *N* (%)	Childbearing wish *N* (%)	Successful conception *N* (%)	Abortions *N* (%)	IVF *N* (%)	Delivery modus
Kwon et al. 2009 [18]	21	26.7	17 (80.9%) : IA 4 (19.04%) : IC	5 (23.8%)	5 (100%)	0	0	Spontaneous
Schlaerth et al. 2009 [3]	20	27	11 (55%) : IA 9 (45%) : IC	6 (30%)	6 (100%)	NR	NR	NR
Borgfeldt et al. 2007 [20]	11	27.5	IA–IC	NR	7	0	NR	NR
Morice et al. 2005 [29]	34	27	30 (88%) : Ia 3 (8.8%) : Ic 1 (3%) : IIa	NR	9	1 (11.1%)	0	NR
Schilder et al. 2002 [11]	52	26	42 (80.7%) : IA 10 (19.2%) : IC	24 (46.1%)	17 (71%)	5 (29%)	0	NR
Morice et al. 2001 [30]	25	24	19 (76%) : IA 6 (24%) : IC	4 (16%)	4 (100%)	1 (25%)	NR	NR
Raspagliesi et al. 1997 [31]	10	22.7	Ia,G3 : 2 (20%) Ic : 2 (20%) IIIa : 2 (20%) IIIc : 4 (40%)	5 (50%)	3 (60%)	1 (33%)	0	NR
Zanetta et al. 1997 [12]	56	29	32 (57%) : IA 2 (3.5%) : IB 22 (39%) : IC	NR	20	4 (20%)	0	15 (75%): vaginal 2 (10%): caesarean sections
Satoh et al. 2010 [16]	211	29	126 (60%) IA 85 (40%) IC	84 (40%)	55 (66%)	10 (18%)	5 (2.4%)	NR

Total	440	28	279 (63.4%) : IA 4 (1%) : IB 141 (32%) : IC		127 (29%)	22 (17.3%)	5	Mainly spontaneous

NR: not reported.
